# How Patients With Cancer Use the Internet to Search for Health Information: Scenario-Based Think-Aloud Study

**DOI:** 10.2196/59625

**Published:** 2025-01-16

**Authors:** Fiorella Huijgens, Pascale Kwakman, Marij Hillen, Julia van Weert, Monique Jaspers, Ellen Smets, Annemiek Linn

**Affiliations:** 1 Department of Medical Psychology Amsterdam University Medical Centers, location Academic Medical Center University of Amsterdam Amsterdam Netherlands; 2 Quality of Care Amsterdam Public Health Amsterdam Netherlands; 3 Cancer Treatment and Quality of Life Cancer Center Amsterdam Amsterdam Netherlands; 4 Amsterdam School of Communication Research/ASCoR Department of Communication Science University of Amsterdam Amsterdam Netherlands; 5 Digital Health Amsterdam Public Health Amsterdam Netherlands; 6 Department of Medical Informatics Amsterdam University Medical Centers, location Academic Medical Center University of Amsterdam Amsterdam Netherlands; 7 Health Behaviors and Chronic Diseases Amsterdam Public Health Amsterdam Netherlands

**Keywords:** web-based health information seeking, think aloud, scenario based, cancer, patient evaluation, information seeking, web-based information, health information, internet, pattern, motivation, cognitive, emotional, response, patient, survivor, caregiver, interview, scenario, women, men

## Abstract

**Background:**

Patients with cancer increasingly use the internet to seek health information. However, thus far, research treats web-based health information seeking (WHIS) behavior in a rather dichotomous manner (ie, approaching or avoiding) and fails to capture the dynamic nature and evolving motivations that patients experience when engaging in WHIS throughout their disease trajectory. Insights can be used to support effective patient-provider communication about WHIS and can lead to better designed web-based health platforms.

**Objective:**

This study explored patterns of motivations and emotions behind the web-based information seeking of patients with cancer at various stages of their disease trajectory, as well as the cognitive and emotional responses evoked by WHIS via a scenario-based, think-aloud approach.

**Methods:**

In total, 15 analog patients were recruited, representing patients with cancer, survivors, and informal caregivers. Imagining themselves in 3 scenarios—prediagnosis phase (5/15, 33%), treatment phase (5/15, 33%), and survivor phase (5/15, 33%)—patients were asked to search for web-based health information while being prompted to verbalize their thoughts. In total, 2 researchers independently coded the sessions, categorizing the codes into broader themes to comprehend analog patients’ experiences during WHIS.

**Results:**

Overarching motives for WHIS included reducing uncertainty, seeking reassurance, and gaining empowerment. At the beginning of the disease trajectory, patients mainly showed cognitive needs, whereas this shifted more toward affective needs in the subsequent disease stages. Analog patients’ WHIS approaches varied from exploratory to focused or a combination of both. They adapted their search strategy when faced with challenging cognitive or emotional content. WHIS triggered diverse emotions, fluctuating throughout the search. Complex, confrontational, and unexpected information mainly induced negative emotions.

**Conclusions:**

This study provides valuable insights into the motivations of patients with cancer underlying WHIS and the emotions experienced at various stages of the disease trajectory. Understanding patients’ search patterns is pivotal in optimizing web-based health platforms to cater to specific needs. In addition, these findings can guide clinicians in accommodating patients’ specific needs and directing patients toward reliable sources of web-based health information.

## Introduction

### Background

Patients with cancer increasingly use web-based platforms to seek information about their diagnosis, treatment, and implications thereof in the short and long term. In the Netherlands, 85% of patients with cancer use the internet [1,2], a rate comparable to that in most Asian countries [3] and other European countries [4,5]. The internet offers a wealth of information that can be readily accessed. It provides practically limitless opportunities for finding health information and support from both lay and expert perspectives, making it a highly popular source of information for many patients.

Within the context of cancer, patients’ web-based health information seeking (WHIS) behaviors have been explained through theories of coping behavior. Most often, cancer literature on information-seeking patterns revolves around coping behaviors such as monitoring and blunting. Studies suggest that most patients manage health threats by proactively seeking information, a behavior referred to as monitoring coping style, whereas others choose to avoid information and opt for distraction, known as blunting coping style [6,7]. However, some studies indicate that the WHIS behaviors of patients with cancer could be explained via a broader range of approaches than merely through theories of coping behavior [8-10]. For instance, patients with cancer could also differ in their choices regarding the kind, quantity, and origins of the sought information, as well as the strategies used for information management. These approaches are based on patients’ perceptions of self-care, which means that patients vary in their WHIS based on what they need to adequately take care of themselves [10]. In addition, the reasons behind seeking information and emotional support on the web are contingent on how patients use the internet [9].

Another factor that could explain variations in how people use the internet is patients’ disease and treatment stage—which may predict different needs concerning the type and amount of information [11,12]. However, studies investigating WHIS and particularly the motives to engage in WHIS often treat the behavior as a one-time event. By treating WHIS as a one-time event, researchers tend to overlook the dynamic nature of health information needs and fail to capture the evolving motivations that patients experience throughout their disease trajectory. Considering that searching for health information is a rather longitudinal behavior, especially for patients moving through different stages of the disease trajectory, a longitudinal lens is required when studying WHIS [11].

In addition to the different phases in the disease trajectory influencing how patients use the internet, WHIS may also vary depending on patients’ *motives* for going on the web. For example, patients may do so to address their cognitive (ie, the need for understanding) and affective (ie, the need to be understood) needs [13]. Cognitive needs (eg, engaging with the internet to enhance preparedness and comprehension of the information provided during a consultation or to validate or challenge the information offered by the provider) will lead to diverse forms of WHIS compared to affective needs (eg, using the internet for peer interaction). In other words, patients’ specific goals regarding information seeking could also impact their search queries [13]. However, these motives are often not sufficiently taken into account when studying WHIS behavior.

Finally, in the period between diagnosis and cure or remission, patients often experience a range of emotions, including (but not limited to) uncertainty, hope, fear, and anxiety. These feelings and emotions are important motivators for many patients to seek out information to cope with their illness [14]. For example, when just diagnosed with cancer, individuals might be concerned about the unpredictable aspects of the disease, leading them to search for information to better manage and cope with their newly discovered illness. Apart from instigating patients’ WHIS behavior, these emotions may also influence decisions to continue, expand, or terminate WHIS [10,14-16]. Earlier qualitative studies have identified various WHIS patterns and the emotions associated with them, ranging from intense to guarded information seeking [10,16,17]. While all participants in these studies expressed a desire for basic information about their diagnosis, they also exhibited diversity in their motivations for seeking cancer information; the emotions experienced; and the nature, quantity, and sources of the sought information, along with the strategies used to manage this information. However, interviews rely on patients’ subjective, retrospective reporting and, therefore, do not provide a comprehensive overview of WHIS behavior.

Hence, it is thus far largely unknown how various motives and emotions guide WHIS behavior in various phases of the cancer disease trajectory, whereas such insights can lead to better designed web-based health platforms catering to patients’ changing requirements and supporting them effectively throughout their health journey. In addition, having a comprehensive understanding of how patients navigate information acquisition on the internet is crucial for establishing effective patient-provider communication that accommodates patients’ specific needs. These insights may also make health care providers aware of the potential impact that WHIS has on patients and, consequently, on the consultation.

### Objectives

Studying the impact of motives and emotions on information-seeking behavior during the disease trajectory poses several challenges that have not been taken into account in previous studies. First, as most WHIS occurs in private settings, such as at home, most of these studies use data collection methods that rely on patients’ subjective, retrospective reporting, such as surveys, focus groups, and interviews. Using these retrospective methods presents significant drawbacks, including recall bias, which may lead to inaccurate results [18]. In particular, information collected before or during diagnosis is considered challenging as this often entails a short and stressful period for many patients [19]. New research methods such as the think-aloud method enable participants to verbalize what they are thinking and doing while performing a certain task [20]; this allows researchers to observe patients’ WHIS more precisely. This includes assessing attention to web-based information, choices made while selecting information, and people’s thoughts and feelings evoked during exposure to information [21]. When combining the think-aloud method with vignettes representing different scenarios at various stages of the disease trajectory, research has the potential to provide a more comprehensive and naturalistic view on the WHIS of patients with cancer. Therefore, this study aimed to explore patterns of motivations and emotions behind the web-based information seeking of patients with cancer at different stages of their disease trajectory, as well as the cognitive and emotional responses evoked via a scenario-based, think-aloud approach. This study adopted a unique explorative approach by observing analog patients (ie, patients or healthy participants putting themselves in the position of a patient [22]) as they engaged in WHIS during different phases of their disease trajectory.

## Methods

### Study Design, Setting, and Population

We used a scenario-based, think-aloud approach followed by a semistructured interview to obtain more in-depth information regarding analog patients’ search strategy, their reasoning and emotions behind this strategy (ie, motives), and the emotions experienced throughout. To increase feasibility and for ethical reasons, we decided to rely on analog patients (patients or healthy participants who are asked to imagine themselves in the role of the patients), who are considered valid proxies for clinical patients [23,24]. The COREQ (Consolidated Criteria for Reporting Qualitative Research) guidelines were used to report the methods (Multimedia Appendix 1).

Analog patients were recruited from a local panel of patients with cancer, survivors, and their informal caregivers who were willing to participate in scientific research on patient-provider communication and health information provision [25]. In this way, we ensured that the analog patients had some personal experience with cancer. Via email, panel members were informed about the study purpose and invited to complete a screening questionnaire to establish their eligibility, that is, whether they were aged ≥18 years, had previously used the internet to search for health information, and owned a computer or laptop with internet connection. The screening questionnaire also included panel members’ age, gender, and educational attainment to allow for purposive sampling based on these characteristics as research shows that individuals differing in these characteristics navigate the web differently and differ in information needs [26]. In addition, we strived for diversity in relation to cancer experience (eg, “I have (had) cancer” or “My partner has (had) cancer”), cancer type, and frequency of using the internet for health information in the previous year (eg, “1-5 times,” “6-10 times,” “11-30 times,” or “more than 30 times”).

In total, 75 panel members indicated an interest in participating. Of these 75 members, we invited 34 (45%) individuals based on purposive sampling to take part in the scenario-based, think-aloud study. Eventually, of the 34 individuals, 5 (15%) participated in the pilot study, and 15 (44%) participated in the think-aloud sessions, 5 (33%) for each scenario. Among the 34 individuals, there were 9 (26%) nonresponses, 1 (3%) failed recording, and 4 (12%) who opted out.

### Procedure

The scenario-based, think-aloud sessions were conducted between May 2021 and December 2021 by 3 researchers (PK, FH, and an undergraduate student). PK and the student have a health communication background, and FH has a health science and health care management background. PK is trained in qualitative research. Due to the COVID-19 pandemic, the sessions were held on the web using videoconferencing software (ie, Zoom [Zoom Video Communications] or Microsoft Teams [Microsoft Corp]) and were recorded with video. Analog patients could participate in the sessions from the comfort of their home while using their own devices, thereby enhancing ecological validity.

We used a protocol for the scenario-based, think-aloud sessions, including a semistructured interview guide. This protocol was pilot-tested with 15% (5/34) of the analog patients. On the basis of the pilot, we decided to develop a video tutorial explaining the think-aloud procedure and a written manual explaining the use of the videoconferencing software (eg, “How do I share my screen?”). We also adapted the interview guide by adding questions focusing on analog patients’ explanations of and reflections on their WHIS behavior (Multimedia Appendix 2). Participating analog patients received an email including an information letter and the video tutorial.

At the start of each session, the researcher explained the nature of the scenario-based, think-aloud method to the analog patients and asked for their personal experience with WHIS. Then, to become familiar with the process of thinking aloud, the analog patients were presented with a practical task (ie, to find a recipe for a pie or a cake containing apples) [27].

After familiarizing the analog patients with the think-aloud procedure, the researcher asked them to imagine themselves in one of the three following scenarios: (1) being an individual who experienced symptoms that could point toward non-Hodgkin lymphoma (NHL), hereinafter referred to as analog prediagnostic patient; (2) being a patient who is about to receive treatment for NHL, hereinafter referred to as analog patient with cancer; or (3) being a survivor of NHL 2 months after having finished treatment, hereinafter referred to as analog survivor of cancer (Multimedia Appendix 3). We use the general term *analog patients* when referring to 2 or 3 scenarios.

Each scenario was based on real patient experiences that were reported in blogs and discussion groups of the largest cancer-related website in the Netherlands [28] and was reviewed by a survivor of cancer to optimize external validity [29]. Analog patients were assigned to the scenario that was most appropriate given their health status and relationship to cancer.

To enhance identification, analog patients were asked to report in their own words what they had just heard in the scenario. In addition, the researcher asked analog patients to discuss any thoughts or feelings that were evoked by the scenario and score their stress, anxiety, worries about cancer, hope, and uncertainty on an 11-point thermometer-style scale (0=*not at all*; 10=*an extreme amount*). Next, analog patients were asked to go on the web imagining themselves as the described patient in the scenario. While performing the various tasks, analog patients were asked to share their screen. The researcher instructed analog patients to indicate when they wanted to stop their web-based search. If analog patients fell silent during the session, the researcher reminded them to voice their thoughts.

After the think-aloud process, a short semistructured interview was conducted in which the researchers probed for analog patients’ motives (eg, what made them choose particular search terms or why they decided to end their search) and their satisfaction with the content (Multimedia Appendix 2). Each interview session ended with a questionnaire assessing the analog patients’ coping style (Dutch Threatening Medical Situations Inventory [30,31]), uncertainty intolerance (Dutch version of the short Intolerance of Uncertainty Scale [32]), information needs [33], and eHealth literacy (Dutch eHealth Literacy Scale [34]). These measures were used to be able to describe the sample.

### Data Analysis

In total, 2 coders (FH and PK) first familiarized themselves with the data by watching the recordings and reading the interviewer field notes. Second, they independently selected and transcribed parts of each recording that seemed relevant to the research questions (eg, motives and emotions related to WHIS and search strategies). During the analysis, they focused on the analog patients’ actions (observations), their verbalized thoughts during the scenario-based, think-aloud process (what they did vs what they said), and their reflections (interview). What was considered relevant was first discussed with a third team member (AL). Third, the coders independently double coded all relevant fragments. Fragments were coded inductively based on the sensitizing concepts as discussed in the introduction (ie, emotions and motives to seek web-based health information, search strategy used, and type of emotions evoked). During the observations, the coders closely examined the search terms used by the analog patients and the content viewed to deduce the analog patients’ underlying motives. Fourth, the coders met and discussed their codes after each session to reach an agreement on the coding scheme together with a third team member (AL). Fifth, after completion of the coding process, the codes were aggregated into potential overarching themes and subthemes through comparisons and discussion between the coders. To improve reliability, validity, and generalizability, the results were substantiated using vivid quotes, and a continuous process of reflection and discussion among the coauthors (FH, PK, AL, and ES) was used. To improve the readability of the overall analysis (N=15), we decided to use the term *most* when the analysis applied to >10 analog patients, *several* when it applied to between 5 and 10 analog patients, and *some* when the analysis applied to <5 analog patients. For scenario-specific analysis (5/15, 33%), we decided to use the term *most* when the analysis applied to 3 or 4 analog patients and the term *some* when the analysis applied to 2 analog patients.

### Ethical Considerations

The Amsterdam School of Communication Research Ethical Review Board approved this study at the University of Amsterdam (ethics approval code: 2021-PC-13493). Informed consent was verbally obtained from analog patients at the start of the scenario-based, think-aloud session. Analog patients could withdraw their consent at any time. The data could not be anonymized as the think-aloud interviews were video recorded. The data are saved on a secured drive of the Amsterdam University Medical Center. No compensation was provided to the participants.

## Results

### Sample Characteristics

Among the 15 participating analog patients (n=9, 60% women and n=6, 40% men), the ages ranged from 28 to 72 years (mean 56.9, SD 12.5 years). Most were former patients with cancer and reported having used the internet for seeking health information >6 times in the foregoing year. In total, the sessions lasted between 25 and 70 minutes, and the web-based search lasted between approximately 6 and 26 minutes. The number of web pages visited ranged from 3 to 15 per session, and changes in search terms ranged from 1 to 16 per session. Table 1 shows the sample characteristics, and Tables 2-4 provide descriptions of the individual search sessions.

**Table 1:** Analog patient characteristics (N=15).

			Prediagnosis stage (n=5)		Treatment stage (n=5)		Survivor stage (n=5)		Total
**Age (y), mean (SD; range)**			59.6 (8.1; 51-72)		54.6 (14.9; 28-63)		56.4 (15.7; 29-66)		56.9 (12.5; 28-72)
**Gender, n (%)**									
	Woman	3 (60)		3 (60)		3 (60)		9 (60)	
	Man	2 (40)		2 (40)		2 (40)		6 (40)	
**Educational level, n (%)^a^**									
	Low	1 (20)		1 (20)		1 (20)		3 (20)	
	Middle	0 (0)		1 (20)		2 (40)		3 (20)	
	High	4 (80)		3 (60)		2 (40)		9 (60)	
**Relationship to cancer, n (%)**									
	Having cancer	0 (0)		2 (40)		1 (20)		3 (20)	
	Having had cancer	2 (40)		3 (60)		4 (80)		9 (60)	
	Having a relative with cancer	3 (60)		0 (0)		0 (0)		3 (20)	
**Frequency of web-based health information seeking in the previous year, n (%)**									
	1-5 times	3 (60)		2 (40)		1 (20)		6 (40)	
	6-10 times	1 (20)		0 (0)		2 (40)		3 (20)	
	11-30 times	0 (0)		3 (60)		1 (20)		4 (27)	
	>30 times	1 (20)		0 (0)		1 (20)		2 (13)	
**Uncertainty intolerance score, mean (SD; range)**			36.2 (7.9; 25-46)		31.8 (9.4; 24-47)		25.6 (7.4; 15-36)		31.2 (8.9; 15-47)
**eHEALS^b^ score, mean (SD; range)**			34.6 (3.8; 31-40)		34.0 (5.3; 27-40)		36.6 (2.1; 34-39)		35.1 (3.8; 27-40)
**Monitoring coping style score, mean (SD; range)**			11.8 (2.6; 8-15)		13.0 (2.3; 10-15)		8.2 (1.3; 6-9)		11.0 (2.9; 6-15)
**Information preference, n (%)**									
	“I want to know as much as possible, both positive and negative information.”	4 (80)		4 (80)		3 (60)		11 (73)	
	“I want to know as much as possible, both positive and negative information, but in a dosed way (little by little).”	1 (20)		1 (20)		1 (20)		3 (20)	
	“I want mainly positive information.”	0 (0)		0 (0)		1 (20)		1 (7)	
	“I don’t need to know that much*.*”	0 (0)		0 (0)		0 (0)		0 (0)	

^a^Low: secondary education; middle: senior general secondary education, secondary vocational education, and preuniversity education; high: college or university.

^b^eHEALS: eHealth Literacy Scale.

**Table 2:** Characteristics of the participants and search sessions in the prediagnosis phase.

		Participant S01	Participant S05	Participant S06	Participant S08	Participant S10	Values, mean (SD)
**Age (y)**		61	51	54	60	72	59.6 (8.1)
**Gender**		Man	Woman	Man	Woman	Woman	—^a^
**Educational level^b^**		High	High	High	Low	High	—
**Search time**		15 min 52 s	8 min 57 s	16 min 51 s	6 min 11 s	7 min 55 s	11 min 9 s (4 min 51 s)
**Times changing search terms, N**		9	4	8	4	1	5.2 (3.3)
**Search engine used**		Google	Google	Google	Google	Google	—
**Total web pages visited, N**		5	9	9	3	5	6.2 (2.7)
**Uncertainty intolerance score^c^**		35	34	41	46	25	36.2 (7.9)
**eHealth literacy score^d^**		33	32	37	31	40	34.6 (3.8)
**Monitoring coping style score^e^**		11	8	15	12	13	11.8 (2.6)
**Thermometer score^f^**							
	Feelings of stress and anxiety	7	6.5	8	7	5	6.7 (1.1)
	Worries about cancer	7	7.5	6	5.5	5	6.2 (1.0)
	Hope	—	—	—	—	—	—
	Uncertainty	7	7.5	8	6	10	7.7 (1.5)

^a^Not applicable.

^b^Low: secondary education; middle: senior general secondary education, secondary vocational education, and preuniversity education; high: college or university.

^c^Uncertainty intolerance was measured using the Intolerance of Uncertainty Scale. Total sum scores can range from 12 to 60. A higher score indicates a higher level of intolerance of uncertainty.

^d^Self-perceived eHealth literacy was measured using the eHealth Literacy Scale (eHEALS). Scores on the eHEALS are summed and can range from 8 to 40, with higher scores representing higher self-perceived eHealth literacy.

^e^Monitoring coping style was measured using the Threatening Medical Situations Inventory. Scores are based on the sum of items and can range from 3 to 16. A higher score means a higher monitoring coping style.

^f^Analog patients were asked before the search session to score feelings of stress and anxiety (thermometer 1), worries about cancer (only for the first scenario), hope (only for the second and third scenarios; thermometer 2), and uncertainty (thermometer 3) on an 11-point thermometer-style scale (*0=not at all*; *10=an extreme amount*).

**Table 3:** Characteristics of the participants and search sessions in the treatment phase.

		Participant S23	Participant S24	Participant S25	Participant S27	Participant S28	Values, mean (SD)
**Age (years)**		61	62	63	59	28	54.6 (14.9)
**Gender**		Woman	Man	Woman	Woman	Man	—^a^
**Educational level^b^**		Low	High	High	Middle	High	—
**Search time**		9 min 55 s	13 min 40 s	16 min 34 s	24 min 55 s	16 min 35 s	16 min 19 s (5 min 31 s)
**Times changing search terms, N**		5	5	9	11	9	7.8 (2.7)
**Search engines used**		Google	Google and Microsoft Bing	Google, Firefox, and Norton Safe Search	Google	Google	—
**Total web pages visited, N**		5	3	10	11	9	7.6 (3.4)
**Uncertainty intolerance score^c^**		32	32	24	47	24	31.8 (9.4)
**eHealth literacy score^d^**		36	40	30	36	27	34 (5.3)
**Monitoring coping style score^e^**		15	15	10	14	11	13 (2.3)
**Thermometer score^f^**							
	Feelings of stress and anxiety	7	8	7	9	8	7.8 (0.8)
	Worries about cancer	—	—	—	—	—	—
	Hope	9	3	9.5	4	4.5	6 (3.0)
	Uncertainty	8	8.5	2	9	5.5	6.6 (2.9)

^a^Not applicable.

^b^Low: secondary education; middle: senior general secondary education, secondary vocational education, and preuniversity education; high: college or university.

^c^Uncertainty intolerance was measured using the Intolerance of Uncertainty Scale. Total sum scores can range from 12 to 60. A higher score indicates a higher level of intolerance of uncertainty.

^d^Self-perceived eHealth literacy was measured using the eHealth Literacy Scale (eHEALS). Scores on the eHEALS are summed and can range from 8 to 40, with higher scores representing higher self-perceived eHealth literacy.

^e^Monitoring coping style was measured using the Threatening Medical Situations Inventory. Scores are based on the sum of items and can range from 3 to 16. A higher score means a higher monitoring coping style.

^f^Analog patients were asked before the search session to score feelings of stress and anxiety (thermometer 1), worries about cancer (only for the first scenario), hope (only for the second and third scenarios; thermometer 2), and uncertainty (thermometer 3) on an 11-point thermometer-style scale (0=*not at all*; 10=*an extreme amount*).

**Table 4:** Characteristics of the participants and search sessions in the survivor phase.

		Participant S32	Participant S34	Participant S35	Participant S36	Participant S37	Values, mean (SD)
**Age (y)**		66	63	66	29	58	56.4 (15.7)
**Gender**		Woman	Man	Man	Woman	Woman	—^a^
**Educational level^b^**		Low	High	High	Middle	Middle	—
**Search time**		15 min 11 s	21 min 40 s	8 min 40 s	25 min 55 s	23 min 35 s	19 min 00 s (7 min 01 s)
**Times changing search terms, N**		6	16	4	13	8	9.4 (5.0)
**Search engines used**		Microsoft Bing	Google and Microsoft Bing	Google	Microsoft Bing	Google	—
**Total web pages visited, N**		8	13	4	15	12	10.4 (4.4)
**Uncertainty intolerance score^c^**		25	26	15	26	36	25.6 (7.4)
**eHealth literacy score^d^**		38	39	34	37	35	36.6 (2.1)
**Monitoring coping style score^e^**		9	6	9	9	8	8.2 (1.3)
**Thermometer score^f^**							
	Feelings of stress and anxiety	5	8	6	3.5	8	6.1 (1.9)
	Worries about cancer	—	—	—	—	—	—
	Hope	3	6.5	8	10	8	7.1 (2.6)
	Uncertainty	7.5	6	0	5	9	5.5 (3.4)

^a^Not applicable.

^b^Low: secondary education; middle: senior general secondary education, secondary vocational education, and preuniversity education; high: college or university.

^c^Uncertainty intolerance was measured using the Intolerance of Uncertainty Scale. Total sum scores can range from 12 to 60. A higher score indicates a higher level of intolerance of uncertainty.

^d^Self-perceived eHealth literacy was measured using the eHealth Literacy Scale (eHEALS). Scores on the eHEALS are summed and can range from 8 to 40, with higher scores representing higher self-perceived eHealth literacy.

^e^Monitoring coping style was measured using the Threatening Medical Situations Inventory. Scores are based on the sum of items and can range from 3 to 16. A higher score means a higher monitoring coping style.

^f^Analog patients were asked before the search session to score feelings of stress and anxiety (thermometer 1), worries about cancer (only for the first scenario), hope (only for the second and third scenarios; thermometer 2), and uncertainty (thermometer 3) on an 11-point thermometer-style scale (0=*not at all*; 10=*an extreme amount*).

### Start of the Search Session

Analog patients reported starting their search session with various associations and reactions evoked by the scenario. For example, in the scenario in which they were experiencing symptoms, analog prediagnostic patients were immediately worried about cancer or felt alarmed by specific symptoms. This was reflected in their search terms, showing a predominant focus on searching for information about these symptoms. This was also reflected in their thoughts as patients expressed concern about the symptoms. Whenever the general practitioner in the scenario showed concern, analog patients more often showed signs of feeling distressed:

The word tumor immediately pops into my mind. This is serious. These are symptoms I would not trust.

S01; analog prediagnostic patient

You do not immediately think the best, especially sweating attacks and weight loss are warning signs.

S05; analog prediagnostic patient

Most analog patients with cancer assigned to the scenario of undergoing cancer treatment started their search by expressing fear about the upcoming challenges, particularly the apprehension of chemotherapy, and harboring doubts about the effectiveness of the treatment. The aggressive nature of NHL added to their anxiety, with a lack of optimistic information causing visible distress and confusion about the treatment process:

I am scared of what’s coming and scared of the chemo. And I am not so hopeful because of my doubt whether the treatment will work.

S27; analog patient with cancer

Despite these negative emotions, some analog patients with cancer still remained combative or hopeful:

Damn, I have cancer again, now I have to have another treatment, but well I am going for it, because I am far from finished living.

S25; analog patient with cancer

This fear was also reflected in their search, with all analog patients with cancer being prone to mainly focus on using search words that were used in the scenario (*(aggressive) non-Hodgkin* and *R-CHOP* [rituximab, cyclophosphamide, hydroxydaunorubicin, oncovin, and prednisone regimen]).

Finally, those who were allocated to the survivor case (“analog survivors of cancer”) generally voiced uncertainty at the beginning of the search about whether the cancer was definitely gone. They showed concerns about cancer recurrence and recovery and were somewhat skeptical about recovery:

Should I really be happy with being cancer-free? What if it comes back? Before this, I had not felt anything. Now, I do not know what I should and should not feel anymore.

S32; analog survivor of cancer

Analog survivors of cancer voiced that, most of all, they wanted to return to their normal lives before the diagnosis and, accordingly, started with search terms related to this desire to get back to the normality of their lives (eg, *out of cancer treatment*, *what now?*).

### Search Motives

#### Overview

**Figure 1:**
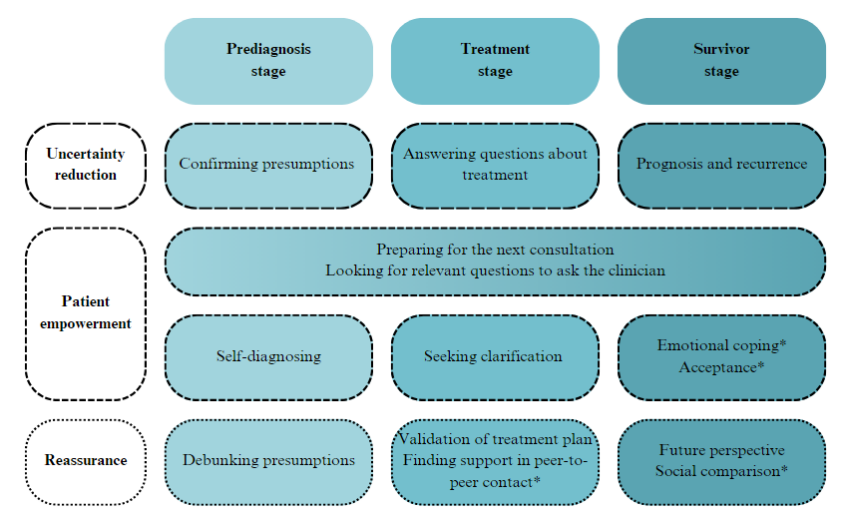
Expressions of the 3 overarching motives for web-based health information seeking—uncertainty reduction, patient empowerment, and reassurance—within the 3 disease stages (prediagnosis stage, treatment stage, and survivor stage). *Affective needs (ie, need to be understood).

On the basis of the search strategies observed and the thoughts voiced, we were able to distinguish 3 overarching motives guiding patients’ WHIS. These overarching motives were prevalent regardless of the allocated stage in the disease trajectory. Each overarching motive was expressed differently throughout the various disease stages (Figure 1). The first motive was *uncertainty reduction* to cope with the anxiety and health threats as most analog patients started their search by expressing uncertainty about what was going to happen to them. The second motive was *empowerment* (ie, “the process of increasing the capacity of individuals (or groups) to make choices and to transform those choices into desired actions and outcomes” [35]) as most analog patients searched content to pursue an active role in their own care process, for example, by actively preparing for the next consultation and looking for relevant questions to ask the clinician. The third motive was *finding reassurance* as analog patients wished to find content that would give them some hope. The 3 overarching motives were not mutually exclusive; they could go hand in hand.

#### Motives in the Prediagnosis Stage

Analog prediagnostic patients wanted to diminish their anxiety and reduce their uncertainty by starting their search with confirming their presumptions. One analog prediagnostic patient immediately used the search term *characteristics cancer*, looked on different websites to compare symptoms, and said the following at search onset:

You do not know anything for sure...Apart from the fact that I initially think it is cancer, I still want to confirm that by searching the internet.

S10; analog prediagnostic patient

Furthermore, they mostly used the internet to empower themselves by attempting to self-diagnose and prepare for the next consultation. When trying to self-diagnose, they used symptom-related search terms, such as *fatigue*, *swollen glands*, *(unexplained) weight loss*, and *night sweats*. After encountering content about possible diagnoses, some changed their search terms to *symptoms of non-Hodgkin* and *symptoms of cancer* while simultaneously explaining this change:

I am actually finding several causes now and cancer is also mentioned. However, I am not quite happy with the information I’m getting yet. But since cancer has come up a few times, I am going to search for symptoms of cancer, so I’m turning it [the search terms] around now [searches for: symptoms of cancer].

S06; analog prediagnostic patient

The motive *empowerment* was apparent in one analog prediagnostic patient who used the search terms *preparing consult internist* and read the text *What can you do to prepare for the first visit with an internist?*:

What I would do now, because I am going to the hospital, is that I am going to prepare. So, I am now going to search on prepare consult internist. I think I am going to an internist, but obviously I’m not sure yet. [reads text on how to prepare for a visit] I would also like to know, what are useful questions? [clicks on other website] Okay, I have pretty much got everything now I need to consider, only I have to go through the 3 good questions again which I can ask the internist [opens the online brochure about 3 good questions].

S06; analog prediagnostic patient

The extent to which analog prediagnostic patients in this phase narrowed down their search to know their exact (possible) diagnosis differed. Some searched various options related to the symptoms, one settled for the likely diagnosis “cancer,” whereas others continued their search until they had a specific idea about the type of cancer. Those who searched for various possible diagnoses wanted to be reassured that the symptoms could be anything other than a serious illness such as cancer. They tried to debunk their presumptions, as reflected in the following observation and quote:

[reads content about causes of swollen lymph nodes] Infection, which could also be, that makes sense. Then I see here swollen nodes due to a systemic disease. Then I am thinking about Lyme disease, okay. That is different from a tumor. Autoimmune disease is potentially on the table. I already see that swollen nodes can be caused by many factors, which is somewhat reassuring.

S01; analog prediagnostic patient

#### Motives in the Treatment Stage

Analog patients with cancer mostly appeared to use the internet to answer their remaining questions to reduce uncertainty. Reducing uncertainty seemed to be combined with increasing their feeling of empowerment as they appeared to seek for more clarification about diagnosis and treatment. Both uncertainty reduction and empowerment were reflected in search terms such as *What is non-Hodgkin lymphoma?*, *R-CHOP*, *side effects*, and *immunotherapy* (ie, cognitive needs). While searching these terms, they said the following:

More than 50% of patients with an aggressive non-Hodgkin lymphoma in an advanced stage are cured after treatment with R-CHOP. Okay, that is quite a lot. But, hmm, yes, the other half does not. It is not clear to me whether the half that does not recover remains chronically ill or simply succumbs to death. I would like to know that in that sense.

S28; analog patient with cancer

The motive to obtain reassurance via web-based information was reflected in analog patients with cancer using the internet to validate whether the treatment (as being proposed in the scenario) was indeed the right treatment for them. They specifically searched for websites and information that would convince them of this treatment being the best option:

And I would definitely, before starting that treatment, request a second opinion from another institution to ensure that I...um...yes, receive the correct diagnosis or the right treatment [searches for other hospitals].

S24; analog patient with cancer

One analog patient with cancer also seemed to use the internet to obtain reassurance via socioemotional content. This was reflected in the search term *experiences with R CHOP*. Of note, none of the analog patients with cancer used search terms indicating a need to know more about the prognosis of NHL.

#### Motives in the Survivor Stage

Analog survivors of cancer seemed to use the internet to reduce uncertainty only to a limited extent. When they used the internet for that purpose, they wanted to know more about prognosis and recurrence, as reflected in search terms such as *prognosis*, *late effects*, and *what to expect*. While using these search terms, they said the following:

Yes, you are quite uncertain about how everything will unfold. There are still quite a few questions, and that diminishes over time, but especially in the beginning after that hospital period, you still have quite a lot of questions.

S37; analog survivor of cancer

Analog survivors of cancer mainly used the internet to search for socioemotional content related to pursuing an active role in their own recovery (ie, patient empowerment). This was reflected in search terms regarding feelings, experiences, and emotions (eg, *uncertainty after cancer* and *feelings after non-Hodgkin treatment*). Pursuing an active role in their own recovery mainly encompassed (emotional) coping and finding acceptance (eg, returning to their normal life before diagnosis). Apparently, to satisfy these motives, they often visited blogs of survivors of cancer writing about feelings and experiences and providing advice on coping with survivorship (eg, *how to deal with emotions/fatigue/work/daily life*). Some searched for psychologists or for recovery programs offered by patient organizations or hospitals, which could also be seen as an expression of empowerment:

Not because I do not trust my own hospital, but I just want to look further. What do other hospitals offer their patients? Is there anything I can take advantage of?

S32; analog survivor of cancer

To a lesser extent, analog survivors of cancer went on the web to seek reassurance about their future. They seemed to be reassured when encountering people with similar experiences. For example, one survivor stated the following:

Okay, I found something here, there are more people like me. Shared sorrow is half sorrow.

S34; analog survivor of cancer

### Overall WHIS Patterns

The web-based source that analog patients eventually selected seemed to depend on their cancer-specific knowledge, cancer-related experience, and search experience. The use of cancer-specific knowledge and experience was reflected in selecting familiar and well-known websites about cancer. The use of search experience was reflected in analog patients using strategies that they reported to prefer (eg, preferring to use the search bar on specific websites instead of the regular search engine or the other way around). Analog patients mentioned different reasons for selecting content. The most prevalent reasons were familiarity with a website or organization (eg, the Dutch Cancer Society) or previous experience with a website. Some also mentioned that they selected certain websites as part of habitual behavior rather than for specific reasons. Notably, analog patients also visited websites while voicing doubt about their trustworthiness. It seemed that those analog patients thought that it was more important to find information relevant to fulfill their motives than looking for trustworthy information.

### WHIS Approaches

In total, 2 overarching WHIS approaches could be identified: explorative and focused. Explorative approaches consisted of spontaneously selecting information seemingly without having an explicit information need. Analog patients who used this explorative approach mainly guided their searches by clicking on referral links and using suggestions made by search features on Google, such as the *autocomplete* (a feature within Google Search that makes it faster to complete searches that users start to type. Google’s automated systems generate predictions that help users save time by allowing them to quickly complete the search they already intended to do) and *people also ask* (a feature within Google Search that provides users with additional questions related to their original search query and quick answers to them) functions*.* Analog patients were considered to use a focused approach when they seemed to search more purposefully (ie, mainly selecting information aligned with their verbally expressed specific information needs). For instance, an analog prediagnostic patient searched *symptoms of cancer* and exclusively selected content related to these search terms.

Unlike analog patients using an explorative approach, patients using a focused approach only made use of Google features when these explicitly helped them meet their self-reported information needs. For example, an analog patient with cancer searched for and read information about R-CHOP and subsequently encountered the following suggestions from the Google feature *people also ask*: *What does R-CHOP mean?* and *What is a CHOP cure?*

Several analog patients used both explorative and focused approaches. Some started with a clearly focused search strategy based on an information need but appeared to become emotionally distracted by the encountered content and started to use a more explorative approach. Others started with an explorative approach and were triggered by specific content that led them to adopt a new, more focused approach (eg, understanding difficult, complex words or confirming assumptions). In other words, information needs evolved while searching. WHIS approaches seemed independent of the disease stage that analog patients were allocated to.

### Dissatisfying Content

All analog patients came across dissatisfying content while searching (in other words, content that did not satisfy the wishes of the patients). Examples of dissatisfying content were difficulty navigating systems on websites, cookies, or information not being in line with search motives. When this dissatisfying content was encountered, analog patients most often changed their search terms or quickly moved on to other web pages (the number of web pages visited ranged from 3 to 15 per session). Search terms were frequently changed during a search session (range 1-16 times per session), mostly because of dissatisfying content:

So, I’m not getting anywhere with this either, because I don’t need to know what the cancer looks like...So I guess I’m not getting anywhere with this search term, with the search things. Uhm how am I going to do that?

S35; analog survivor of cancer

### Impact of WHIS on Emotions and Dealing With Content

#### Emotions

Regardless of the stage of the disease, emotions were present throughout the entire search process, ranging from anxiety and worry to hope. These emotions fluctuated, and negative emotions were often induced when confrontational, complex, or unwanted information was found. Confrontational content included information on symptoms suggesting cancer or thyroid problems, information on treatment side effects such as hair loss and nausea, or a confronting picture:

I am not happy with the image I see here. That photo confirms the nightmare I have about chemotherapy. This is someone surrounded by nurses, being injected, and she has no hair, so that picture embodies for me everything that is wrong with this disease in one image. They have succeeded very skillfully in capturing all of that in one photo, but I do not think that was the intention of the person who took the photo. However, that is how it comes across at me: the embodiment of a mountain of misery.

S27; analog patient with cancer

Complex information included content containing medical jargon, such as *malignancies*; *cachexia*; or drug names such as *rituximab*, *cyclophosphamide*, and *hydroxydaunorubicin*. Most analog patients seemed to be affected by complex words:

This is getting annoying because I already see a word here that I do not know at all. I’m getting a lot of medical terms here that do not mean much to me...

S06; analog prediagnostic patient

Sometimes, positive emotions emerged from information that gave hope (eg, indolent NHL more often has a chance of recurrence than aggressive NHL). Moreover, analog patients who doubted their own navigation skills while searching on the web reported high levels of distress. Some of the analog patients also experienced cognitive dissonance (ie, a mental state of having conflicting beliefs, thoughts, values, or attitudes), as reflected in the following quote:

Everything in you says that it is better not to click on it, because you don’t want to know it. But if you see the option then you just need to click on it.

S27; analog patient with cancer

#### Dealing With Emotionally Difficult Content

When encountering cognitively or emotionally difficult (or unwanted) information, analog patients with cancer dealt with the content in various ways. They adapted their search strategy, ignored the information by quickly clicking away from it and shifting toward other information, or stopped searching:

I immediately find myself with types of cancer, um...all the hits are related to Hodgkin; [scrolling back and forth through search results on the first Google page, but not clicking on anything]. Yes, I find this difficult; I think I will check the next Google pages to see what else comes up, what comes after Hodgkin.

S01; analog prediagnostic patient

Several analog patients also mentioned that they would normally seek information multiple times briefly or seek a distraction from the confronting information, such as watching Netflix or having some tea.

### End of the Search

As mentioned previously, one of the reasons to stop searching was encountering cognitively or emotionally difficult information (confronting, upsetting, or confusing). This was mostly the case for analog prediagnostic patients and analog patients with cancer. The following quote illustrates this “overload”:

Nothing [information found] makes me happy. Yeah, you can find information, but I believe I would make a cup of coffee now. I cannot say I’m a lot wiser now.

S25; analog patient with cancer

Another reason to stop searching was that analog patients saw their health care provider as a gatekeeper and their primary source of information about their disease and treatment. During the interview, they indicated that they preferred to talk with their clinician to clarify the encountered information instead of looking for more web-based information:

I believe that this information is quite overwhelming me right now, so I would put it away for a while. And I would talk it through first at a subsequent consultation with my doctor before I start worrying and assuming things that are not an issue at all...So, I think I will stop looking for now until I have spoken to the doctor again. It is a lot of information, and it is also complicated. So, I want to consult the doctor first.

S23; analog patient with cancer

All analog patients with cancer indicated ending their search sessions with many unanswered questions and an increase in uncertainty (compared to the start of the search). Unlike analog patients with cancer, analog prediagnostic patients and analog survivors of cancer ended their search more often with their information needs being fulfilled, as reflected in the following quote during the interview:

I do think it is very true. I’m at a point now where I do think: yeah, I’m reading this now, I’m not really getting very comfortable with this. I do not think there is any point in continuing to search now. I think I am now on a trustworthy site, and I find this a very upsetting story now that I see this. I cannot do much but wait and see. I don’t know if I’m happy I’ve figured this out now...

S01; analog prediagnostic patient

Compared to analog patients in other disease stages, analog survivors of cancer ended their search most often satisfied and with more positive emotions; they felt less uncertain and found useful (practical) information on ways to cope with the future:

I definitely did become a bit wiser, because I can move on: I can go to physio, psychologist and I have a phone line which I can call.

S36; analog survivor of cancer

## Discussion

### Principal Findings

Using a comprehensive scenario-based, think-aloud approach, we were able to show that (1) patients’ overarching motives for WHIS were mainly to reduce uncertainty, obtain reassurance, and increase empowerment; (2) these motives differed depending on the disease stage (at the beginning of the disease trajectory, patients mainly showed cognitive needs, whereas this shifted more toward affective needs in the subsequent disease stages); (3) analog patients’ WHIS approaches varied from exploratory to focused to a combination of both; and (4) positive (hope and reassurance) and negative (anxiety and worry) emotional responses played an important role in patients’ search strategies.

We found 3 overarching motives (ie, reducing uncertainty, obtaining reassurance, and increasing empowerment) for patients to search on the web. With these findings, we not only confirm the problem-solving model in the context of patient motivations to go on the web throughout their illness journey but also extend this model. According to Wilson [36], the process of problem-solving is the result of patients’ wishes to reduce uncertainty. Patients’ uncertainty at either the prediagnosis or treatment phase concerned various topics, clearly showing that these motives change over time. However, we also discovered 2 other important motives for patients to engage in a problem-solving process, namely, reassurance and empowerment [36]. In addition, the study’s findings revealed a potential conflict between patient empowerment and uncertainty reduction in the context of WHIS. When patients seek web-based information to empower themselves, they gain a better understanding of their situation, which could enable them to ask informed questions to their clinicians. However, this increased knowledge may also give rise to new questions and uncertainties, leading to a potential challenge in fulfilling the motive of uncertainty reduction.

Moreover, our findings provide insights into the search behavior of patients with cancer at various stages of their disease trajectory and how these behaviors vary. In the initial phase of prediagnosis, patients often engaged in self-diagnosis. The results of this study extend those of previous research [9] by showing that patients prepare for a consultation by using the internet not only to help them formulate questions but also to self-diagnose. Despite the popularity of this search approach, research on self-diagnosing remains limited. In the context of web-based self-diagnosis for minor ailments, research shows that using the internet for self-diagnosis can be helpful as 44% of participants achieved accurate final diagnoses after searching the internet compared to 11% before searching the internet [37]. Another study shows that web-based self-diagnosing has the potential to empower patients in appraising and challenging clinicians’ advice and requesting further diagnostic procedures [38]. However, web-based self-diagnosis can also be counterproductive if the patient misdiagnoses themselves, leading to unnecessary concerns. In addition, problems may occur if patients visit their clinician with a preconceived diagnosis, potentially causing disagreements about their condition [39]. During the treatment phase, the search strategy of patients with cancer focused on cognitive needs by seeking clarification, gathering more information, and preparing. However, we only observed a shift in search strategies toward affective needs by seeking emotional coping resources for dealing with the disease after patients completed treatments and were declared cancer free. In other words, at the beginning of the disease trajectory, analog patients had mainly cognitive needs, whereas analog survivors also showed affective needs and used the internet for emotional support. The change from more cognitive needs to more affective needs could be explained using the social-cognitive processing model. According to this model, seeking emotional support may facilitate emotional adjustment to traumatic experiences, such as cancer diagnosis and treatment [40]. Potentially, survivors have more mental space to cope with the situation and reflect on what has happened in the past months.

Our results further show that patients tend to use different search strategies: explorative, focused, or a combination of both. Previous research has demonstrated that individuals who are more exploratory seekers tend to tackle unfamiliar problems by using a broader search strategy (symptom exploration), resulting in a broader range of new information [37]. By encountering a broad range of information, patients are possibly confronted with new and unknown content, which could increase their level of uncertainty [41]. Our results also suggest that an exploratory search strategy increased the risk of being confronted with unwanted information. On the other hand, those who are more focused seekers tend to have a clear idea and a specific plan, leading them to research within a limited set of results (hypothesis testing) [37]. Such hypothesis testing can be problematic because it skews the way in which patients process information and distorts their perception of reality—a phenomenon known as confirmation bias [42]. It occurs when patients seek, interpret, or favor information that confirms their existing beliefs while ignoring or downplaying evidence that contradicts those beliefs [43]. Pang et al [41] argue that seekers within one internet visit alternate between exploratory and focused search strategies as new, unknown topics often lead to more exploratory searches. If the topic to be searched becomes clearer, the seeker may use a more focused approach. Our results confirm those of this previous study by showing that patients used both explorative and focused approaches. Some started with a focused search but became emotionally distracted and switched to an explorative approach. Others began exploratively and shifted to a focused search after encountering specific content.

Furthermore, our findings show that positive (hope and reassurance) and negative (anxiety and worry) emotional responses were present before, during, and after the search sessions. On the basis of patients’ voiced thoughts and observed behavior, we conclude that these emotions impacted their search behavior. This is in line with the functionalist perspective of emotions, which argues that emotional responses may motivate people to behave in particular ways [44-46]. For instance, hope is seen as a motivating force that helps individuals move toward desired outcomes even in the face of uncertainty [47]. It is a future-oriented emotion as it involves visualization of positive future situations [48], and thus, hope could explain why patients are motivated to seek reassurance. Worry, on the other hand, is seen as an uncertainty-associated emotion and can increase a patient’s desire for obtaining additional information [15]. Studies show a positive relationship between worry and the perceived need for additional information [49-51], and thus, worry could explain why patients are motivated to reduce uncertainty by searching for additional information. However, we also observed that patients who were worried ignored or avoided specific information. A possible explanation is that hope and worry are intertwined during WHIS [16]. Confronting or complex information poses a threat to hope, and thus, ignoring certain information may serve as a self-protective behavior to stay hopeful [16].

In our study, patients in the treatment phase were most worried after their search session. This is in contrast to existing literature indicating that perceived knowledge through web-based information seeking decreased patients’ worry [15]. WHIS has also been found to help searchers fill information voids and enhance their coping abilities [52]. Although we did find some comparable results for the prediagnosis and survivor phases regarding decrease in worry and enhancing coping abilities, we did not find this for patients in the treatment phase. A possible explanation is that complex or confrontational information (eg, jargon for medicines and treatments and intense side effects) may have induced worries in analog patients in this phase. This inconsistency with the existing literature could further be explained by our design, which involved one search session only at one specific moment rather than multiple search sessions by one individual patient. Possibly, patients who search for more information at multiple times will eventually be less worried as they become more familiar with the difficult and complex information. Therefore, future research should investigate the longitudinal search behaviors of individual patients during their disease trajectory and the effects of multiple shorter search sessions within a particular disease phase.

### Limitations and Strengths

First, a strength of our approach is that we not only observed patients’ WHIS behaviors but simultaneously gained insights into their thoughts. During the interview, the interviewer made use of techniques such as paraphrasing and checking to clarify the meaning of the interviewee, thereby enhancing the validity of our findings. This innovative, comprehensive scenario-based, think-aloud approach exhibits strength in its consideration of the intuitive nature of web-based searching while overcoming challenges such as recall bias in retrospective methods. However, certain limitations should be considered. Some remarks suggested that participants may have felt limited in their choice of search engine and might have perceived an obligation to use a specific search platform, such as Google. Furthermore, during the think-aloud sessions, participants did not explore the use of social media channels (eg, Facebook, Instagram, or Twitter [subsequently rebranded X]). Use of social media may have been limited as participants could perceive it as an intrusion into their personal lives. Another reason could be that these communication channels may represent more spontaneous ways through which patients acquire unplanned or unexpected web-based health information while scrolling through their social media timeline [53]. The scenario-based, think-aloud approach as used in this study does not provide any insights in how social media has an effect on patients’ WHIS strategies, motives, and emotions. Furthermore, the relatively small sample size used in this study calls for caution when generalizing the findings. It is important to account for variations in patients’ (eHealth) literacy, education, and cultural backgrounds [54]. Although previous research demonstrates overlap in WHIS among patients from different countries, it also identifies distinct country-specific differences even when the countries have comparable welfare and health status [5]. As this study was an explorative qualitative study, and despite our relatively small sample size, we believe we achieved thematic saturation during the iterative process as no new codes emerged toward the end of our analysis. Moreover, it is important to bear in mind when interpreting the findings that our sample consisted of analog patients who were presented with a scenario. This may have biased our results as using analog patients is different from using patients with NHL. However, participants in this study possessed preexisting familiarity with cancer; our sample consisted of patients with cancer (other than NHL), survivors of cancer, and informal caregivers of patients with cancer. Thus, this sample’s strength lies in their ability to strongly identify with the scenarios presented, which is also reflected in their quotes, the emotions showed during the think-aloud process, and their scores on the thermometers [24]. Furthermore, participants possessed experience in web-based cancer information seeking. Many of them were acquainted with patient advocacy organizations, and a subset even served as administrators for certain web-based platforms dedicated to cancer information and peer support groups. In addition, they had previously encountered medical terminology in the context of their own medical conditions, thus acquiring a degree of familiarity with medical jargon. Consequently, our sample likely possessed a higher level of proficiency in navigating the internet for cancer-related information compared to the average patient with cancer. Despite their advanced familiarity with the subject, the results still indicated that patients encountered difficulties in navigating the internet and understanding medical jargon.

### Practical Implications

Knowing how patients with cancer search for web-based health information is a first step toward optimizing web-based health platforms such that patients with cancer can (more) easily find and navigate through information that fits their needs. On the basis of the study results, there are various implications for the development of cancer websites. First, web-based health platforms could use less complex words and show content warnings about confrontational prognostic or side effect–related information on web pages. The latter could warn searchers about unwanted information, which is especially relevant for exploratory searchers. Second, websites should enable users to self-pace and allow for user-initiated tailoring (ie, allowing users to tailor the information themselves based on their information needs). For example, information should be minimalized, with the possibility to read more if wanted (eg, with the use of hyperlinks). Third, it should also be clear to the user whether platforms are expert generated or peer generated as these platforms differ in content focusing on cognitive needs (addressing the needs of analog prediagnostic patients and analog patients with cancer) and affective needs (addressing the needs of analog survivors of cancer) [13]. In the Netherlands, multiple cancer platforms already make use of such features, which patients in our sample experienced as convenient. In addition to these implications for websites, another important finding is that patients see their health care providers as their primary source of information when it comes to their disease and treatment. Patients indicated that they had various remaining questions and considerable uncertainty after their search, which they wanted to resolve during their interaction with their health care provider. Therefore, it is important that, within consultations, there is room for questions arising from WHIS. Furthermore, health care providers can guide patients in the search process by giving tips and tricks on how (not) to use the internet to search for health information and how to cope with any uncertainty that may result from such a search.

### Conclusions

This study provides valuable insights into the real-time WHIS strategies of patients with cancer, the motivations behind seeking web-based health information, and the emotions experienced at various stages of the disease trajectory. Understanding patients’ search patterns is pivotal in optimizing web-based health platforms to cater to their specific needs. In addition, these findings can guide clinicians in directing patients toward reliable sources of web-based health information.

## Appendix

Multimedia Appendix 1 COREQ (Consolidated Criteria for Reporting Qualitative Research) checklist.

Multimedia Appendix 2 Final think-aloud protocol, including semistructured interview guide.

Multimedia Appendix 3 Think-aloud scenarios.

## Abbreviations

COREQ: Consolidated Criteria for Reporting Qualitative Research
NHL: non-Hodgkin lymphoma
R-CHOP: rituximab, cyclophosphamide, hydroxydaunorubicin, oncovin, and prednisone
WHIS: web-based health information seeking

## Data Availability

The datasets generated during and analyzed during this study are not publicly available due to the anonymity of the participants but are available from the corresponding author on reasonable request.
