# Supplementary material for: How Patients With Cancer Use the Internet to Search for Health Information: Scenario-Based Think-Aloud Study
Source: JMIR Infodemiology. 2025 Jan 16;5:e59625. doi: 10.2196/59625 (PMC11783026; doi:10.2196/59625)
Supplement: Multimedia Appendix 2 [file infodemiology_v5i1e59625_app2.docx]

## Multimedia Appendix 2. Final think aloud protocol, including semi-structured interview guide

### 1. Practice round to get familiar with talking out loud while searching the internet

Task: Look for a recipe on the internet of a pastry or cake. You have leftover apples, so you would like to use these. The other ingredients are entirely up to you. I would like to ask you to speak your thoughts out loud while searching.

### 2. Introducing the scenario

In advance, the researcher asks the participant to imagine him-/herself in the scenario as best they can. The researcher reads the scenario aloud. The text is then sent to the participant in the Teams chat function so that participant has it ready during the search.

### 3. Checking immediate associations and thoughts

- May I ask you to tell (briefly) in your own words what you have just heard/read?
- After hearing the description, what are the first thoughts and/or feelings that come to mind?
- Score participant’s stress, anxiety, worries about cancer (only for scenario 1), hope (only for scenario 2 and 3) and uncertainty on a 11-point thermometer-styled scale (0 = not at all; 10 = an extreme amount)

### 4. The think aloud session

In case the participant is silent, the researcher will remind him/her to try to express his/her thoughts. In case the participant still does not express thoughts, the researcher can ask the following questions:

- Why do you use these search terms?
- Why are you clicking on this very website?
- How do you like the look of this website?
- What do you think of this?
  - Helpful? Complete? Reliable? Understandable? Disturbing?

### 5. Interview guide after think aloud session

- Why did you decide to stop searching?
- How did you come up with these search terms?
- Thinking about the situation outlined earlier, what were you hoping to find? And why?
- To what extent did the content you encountered meet your expectations?
- What made you use Google/other search engine?
- How difficult or easy was it for you to search on the Internet? Please explain.
- How difficult or easy was it for you to interpret/apply the content you found to your situation? Please explain.
- How do you feel about the content you found?
- To what extent do you think the content you encountered was interesting? Why?
- What kind of content did you encounter that was not interesting for you while searching?
- Would you recommend the found content to a friend in the same situation? Why yes/no?
- What do you think about the amount of content online?
- What do you think of how the content is presented? Think for example about language use and design.
- Do you still miss certain information after searching? If so, what?
