# Supplementary material for: How Patients With Cancer Use the Internet to Search for Health Information: Scenario-Based Think-Aloud Study
Source: JMIR Infodemiology. 2025 Jan 16;5:e59625. doi: 10.2196/59625 (PMC11783026; doi:10.2196/59625)
Supplement: Multimedia Appendix 3 [file infodemiology_v5i1e59625_app3.docx]

## Multimedia Appendix 3. Think aloud scenario’s

### Scenario 1. Pre-diagnosis stage

#### English version

*You recently visited your GP because you have not been feeling very fit for a long time.*

*You have been sick more often and now again it feels like your body is fighting an infection. You no longer think of flu, because despite coughing a lot, you don't feel like you have a cold. When you get up, you stay sleepy for a long time and you continue to feel sluggish during the day too. When you lie in bed at night, you sometimes feel feverish and have sweating attacks. Your fitness seems to have deteriorated as well: when cycling with your friends, you notice that you can't always keep up with them. On top of that, you have lost a lot of weight.*

*The GP also thinks that the symptoms you have are no longer consistent with a flu and noted that your glands in the neck were swollen. The GP has referred you to the hospital and wants your blood tested as soon as possible.*

*You now begin to feel anxious anyway and decide to search online for what the GP could possibly be thinking about.*

#### Dutch version

*Je bent onlangs bij de huisarts geweest omdat je je al een hele tijd niet heel fit voelt.*

*Je bent wel vaker goed ziek geweest en ook nu voelt het alsof jouw lijf vecht tegen een ontsteking. Je denkt niet langer aan griep, want ondanks dat je veel hoest, je bent je niet verkouden. Bij het opstaan blijf je lang slaperig en ook overdag blijf je je futloos voelen. Als je ’s avonds in bed ligt, voel je je soms koortsig en heb je zweetaanvallen. Ook je conditie lijkt achteruitgegaan: als je met je vrienden de fiets pakt om op stap te gaan, merk je dat je ze hen niet altijd meer kan bijbenen. Bovendien ben je ook nog erg afgevallen.*

*De huisarts vindt ook dat de klachten die je hebt niet langer passen bij een griep en merkte op dat je klieren in de hals opgezet waren. De huisarts heeft je doorgestuurd naar het ziekenhuis en wil dat je bloed zo snel mogelijk onderzocht wordt.*

*Je begint je nu toch ongerust te maken en besluit online op zoek te gaan naar waar de huisarts mogelijk allemaal aan kan denken.*

### Scenario 2. Treatment stage

#### English version

*Some time ago, you were told that you have lymphoma, an aggressive non-Hodgkin's lymphoma to be precise. After hearing the diagnosis, your world collapsed. Even though you had the feeling something was wrong, the fear and shock completely overwhelmed you. The doctor saw that you were emotional, and gave you time to let the diagnosis sink in.*

*Last week, you had the follow-up consultation. In it, the doctor explained to you that the cancer is well treatable. The most effective treatment in your case is a combination of chemotherapy and immunotherapy. The doctor also called this treatment R-CHOP.*

*You will start treatment next week, then you will receive the first course of treatment. In total, you will get eight courses, one every three weeks.*

*To get a better grip on everything discussed in the consultation, you decide to look for more information online.*

#### Dutch version

*Enige tijd geleden kreeg je te horen dat je lymfeklierkanker hebt, een agressief non-Hodgkin lymfoom om precies te zijn. Na het horen van de diagnose stortte je wereld in. Ook al had je een voorgevoel, de angst en schrik overrompelden je volledig. De arts zag het aan je, en gaf je de tijd om de diagnose te laten bezinken.*

*Vorige week had je het vervolggesprek. Hierin legde de arts je uit dat de kanker goed te behandelen is. De meest effectieve behandeling in jouw geval is een combinatie van chemotherapie en immunotherapie. De arts noemde dit ook wel behandeling met R-CHOP.*

*Volgende week start je met de behandeling, je zal dan de eerste kuur krijgen. In totaal krijg je acht kuren, om de drie weken één.*

*Om meer grip te krijgen op alles wat er met je besproken is, besluit je online op zoek te gaan naar meer informatie.*

### Scenario 3. Survivor stage

#### English version

*Two months ago, you received your very last chemotherapy treatment for lymphoma. After months of treatments and countless hospital visits, you are done. The doctors had declared you cancer-free; you no longer have non-Hodgkin lymphoma. A huge relief and release followed at first. No more hospital in, hospital out. The treatment process is finished and now only the follow-up checks follow.*

*Now that you are back home, you notice that you are stepping out of survival mode and that there is room for processing. The cancer may be gone, but physically and mentally you are not the same person as you used to be. You notice that your condition has deteriorated considerably and you are still often tired. This makes it difficult to pick up your social life again. You also suffer from concentration problems that get in the way of resuming your work.*

*All this has broken you up considerably over the past week. You wonder how best to deal with this and decide to look for more information online.*

#### Dutch version

*Twee maanden geleden kreeg je jouw allerlaatste chemokuur tegen lymfeklierkanker. Na maanden van behandelingen en talloze ziekenhuisbezoeken was je klaar. De artsen hadden je kankervrij verklaard, je hebt geen non-Hodgkin lymfoom meer. Eerst volgde een enorme opluchting en ontlading. Niet meer ziekenhuis in, ziekenhuis uit. Het behandeltraject is klaar en nu volgen alleen nog de nacontroles.*

*Nu je weer thuis bent merk je dat je uit de overleef-stand stapt en dat er ruimte komt voor verwerking. De kanker mag dan weg zijn, maar je bent zowel fysiek als mentaal niet meer dezelfde persoon als vroeger. Je merkt dat je conditie flink achteruit is gegaan en je bent nog vaak vermoeid. Het is daardoor lastig om je sociale leven weer te herpakken. Ook heb je last van concentratieproblemen die het hervatten van je werk in de weg zitten.*

*Dit alles heeft je de afgelopen week flink opgebroken. Je vraagt je af hoe je hier het best mee om kan gaan en besluit online meer informatie op te zoeken.*
